# Opioid Preconditioning Modulates Repair Responses to Prevent Renal Ischemia-Reperfusion Injury

**DOI:** 10.3390/ph13110387

**Published:** 2020-11-14

**Authors:** Adriana Franco-Acevedo, Raquel Echavarria, Bibiana Moreno-Carranza, Cesar-Ivan Ortiz, David Garcia, Ricardo Gonzalez-Gonzalez, Oscar-Kurt Bitzer-Quintero, Eliseo Portilla-De Buen, Zesergio Melo

**Affiliations:** 1 Doctorado en Farmacologia, Universidad de Guadalajara, Guadalajara 44340, Mexico; ady_francoa@hotmail.com; 2 CONACyT-Centro de Investigacion Biomedica de Occidente, Instituto Mexicano de Seguro Social, Guadalajara 44340, Mexico; rechavarria@conacyt.mx; 3 Escuela de Ciencias de la Salud, Universidad Anahuac Queretaro, Queretaro 76246, Mexico; bibianamorenocarranza@gmail.com; 4 Surgical Research Division, Centro de Investigacion Biomedica de Occidente, Instituto Mexicano de Seguro Social, Guadalajara 44340, Mexico; ci_ortiz_g065@hotmail.com (C.-I.O.); davidga28@gmail.com (D.G.); qfbragg26@gmail.com (R.G.-G.); okbitzer@gmail.com (O.-K.B.-Q.); eportilla@mail.udg.mx (E.P.-D.B.)

**Keywords:** opioids, renal ischemia-reperfusion, morphine, fentanyl, angiogenesis, wound healing

## Abstract

Progression to renal damage by ischemia-reperfusion injury (IRI) is the result of the dysregulation of various tissue damage repair mechanisms. Anesthetic preconditioning with opioids has been shown to be beneficial in myocardial IRI models. Our main objective was to analyze the influence of pharmacological preconditioning with opioids in renal function and expression of molecules involved in tissue repair and angiogenesis. Experimental protocol includes male rats with 45 min ischemia occluding the left renal hilum followed by 24 h of reperfusion with or without 60 min preconditioning with morphine/fentanyl. We analyzed serum creatinine and renal *KIM-1* expression. We measured circulating and intrarenal VEGF. Immunohistochemistry for HIF-1 and Cathepsin D (CTD) and real-time PCR for angiogenic genes *HIF-1α*, *VEGF*, VEGF Receptor 2 (*VEGF-R2*), *CTD*, *CD31* and *IL-6* were performed. These molecules are considered important effectors of tissue repair responses mediated by the development of new blood vessels. We observed a decrease in acute renal injury mediated by pharmacological preconditioning with opioids. Renal function in opioid preconditioning groups was like in the sham control group. Both anesthetics modulated the expression of HIF-1, VEGF, VEGF-R2 and CD31. Preconditioning negatively regulated CTD. Opioid preconditioning decreased injury through modulation of angiogenic molecule expression. These are factors to consider when establishing strategies in pathophysiological and surgical processes.

## 1. Introduction

Renal ischemia-reperfusion injury (IRI) is a pathological process characterized by an initial oxygen perfusion impairment resulting in organ hypoxia and, followed by blood reflow, an oxygenation reestablishment. Renal IRI often follows organ failure, sepsis, hypo/hypertension or surgical procedures, including kidney transplantation [1,2,3]. Regularly, procedure-related hypoxia is exacerbated by the progressive loss of integrity and number of blood vessels (vascular rarefaction) [4,5]. Functional endothelial cells are gradually replaced by tubule-interstitial fibrosis. Chronic hypoxia stimulates the expression of profibrotic molecules such as transforming growth factor beta (TGF-β) [6], contributing to the development of dysfunctional tissue zones.

Microvascular rarefaction after renal IRI promotes chronic organ ischemia, inflammation and progressive loss of organ function [7]. Although the mechanisms involved in the reduction of blood vessels are still unclear, it is known that IRI promotes a reduction in the activity and expression of vascular endothelial growth factor (VEGF) and other molecules associated with the survival and proliferation of endothelial cells. VEGF is one of the most important promoters of angiogenesis, and its expression depends on stimulation by hypoxia-inducible factor 1 alpha (HIF-1α) [8]. It shows proangiogenic function activated through its binding to the vascular endothelial growth factor receptor 2 (VEGF-R2) [9]. HIF-1α is a transcription factor with an essential renoprotective role. Renal activation of HIF-1α promotes the expression of angiogenesis effector proteins such as cathepsin D (CTD) [10] and IL-6 [11]. However, after IRI, its protective activity is repressed [12]. Indeed, IL-6 displays a dual effect on renal ischemia-reperfusion. First, this cytokine can mediate neutrophil activation, one of the central mechanisms for acute kidney injury (AKI) development [13]. On the other hand, it contributes to renal epithelial regeneration following ischemic injury [14] and is an effective proangiogenic molecule, promoting endothelial cell proliferation and migration [15].

The present challenge is to design strategies to reduce renal IRI and to improve patient outcomes. Opioid preconditioning may be a promising strategy, as can be deduced from the successful results obtained after its application in several models of ischemia [16]. Morphine and fentanyl are two opioids commonly used in clinical practice, general surgery and transplantation [17]. Information about the effects of opioids on renal pathophysiology is controversial. Some studies suggest that morphine treatment may cause more intense cisplatin-induced kidney damage [18]. However, there is also evidence showing kidney protection against IRI by the activation of kappa opioid receptors [19]. Therefore, due to its relative safety, some opioids are commonly used to treat pain in patients with end-stage renal disease (ESRD) and kidney transplant procedures [20]. The most studied opioid for analgesia in kidney transplantation is certainly morphine, nevertheless, fentanyl represents a better option with improved pharmacokinetic characteristics and enhanced analgesic response [21].

The participation of opioids in angiogenesis has been well studied [22,23], particularly in cancer and tumor progression [24]. Evidence shows the modulation of molecules such as HIF-1 [25] and VEGF/VEGF-R2 [26] by opioids. Nevertheless, the mechanisms that underlie the renal protective effect of opioid preconditioning have been poorly studied. The purpose of our work was to determine and compare the role of morphine and fentanyl in the modulation of angiogenic molecules that protect the kidney from IRI.

## 2. Results

### 2.1. Opioid Preconditioning Reduced Renal Ischemia-Reperfusion Injury

**Figure 1:**
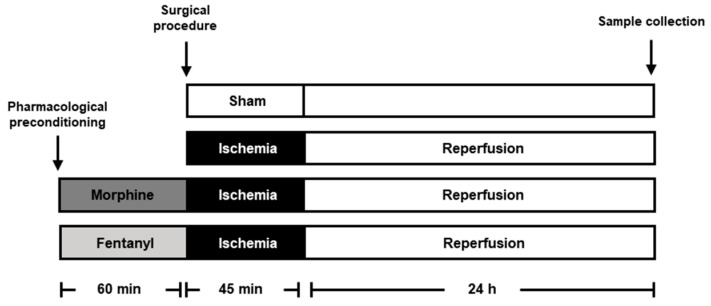
Experimental scheme. Graph illustrating experimental conditions in the study. Six male Wistar rats were treated with 0.5 mg/kg morphine (dark gray bar) or 10 µg/kg fentanyl (light gray bar) for 60 min before the left renal pedicle was occluded for 45 min to promote ischemia and a 24 h reperfusion period. As controls we performed a sham group (white bar) and ischemia-reperfusion injury (IRI) without preconditioning (black bar). The gray scale identification is preserved in all figures. Blood and kidney tissue samples were collected at the end of the reperfusion period.

**Figure 2:**
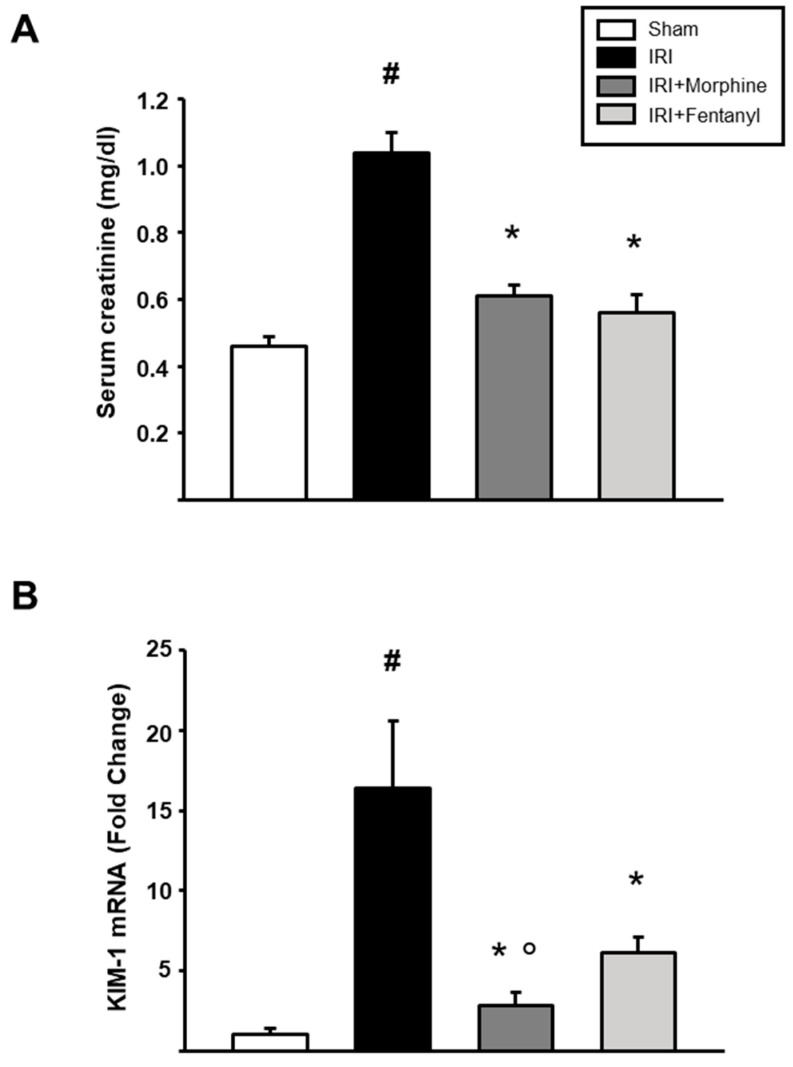
Effects of opioid preconditioning on serum creatinine and *KIM-1* mRNA levels. (**A**) Serum creatinine was evaluated using a dry chemistry technique. (**B**) Renal expression of *KIM-1* was evaluated by quantitative RT-PCR in the ischemia-reperfusion treated kidneys. *HPRT* was used as a housekeeping gene for normalization. The sham group is represented by the white bars, IRI by the black bars, IRI + morphine by the dark gray bars, and IRI + fentanyl by the light gray bars. Values are means ± S.E.M. (*n* = 6). # *p* < 0.05 vs. sham, * *p* < 0.05 vs. IRI, ° *p* < 0.05 vs. fentanyl.

Twenty-four rats were randomly divided into four experimental groups: sham, left unilateral IRI, IRI + morphine, and IRI + fentanyl (Figure 1). To examine the effects of opioid preconditioning on kidney function after IRI, we measured serum creatinine and *KIM-1* in our surgical model. The group of IRI animals showed a significant increase in serum creatinine values when compared to sham animals (1.038 ± 0.0606 mg/dL vs. 0.460 ± 0.0287 mg/dL, respectively, *p* < 0.001; Figure 2A), suggesting the induction of severe AKI. Meanwhile, the preconditioning groups, both morphine and fentanyl, showed a reduction in serum creatinine levels compared to animals subjected to IRI (0.613 ± 0.0324 mg/dL, *p* = 0.002 and 0.562 ± 0.0517 mg/dL, *p* < 0.001, respectively; Figure 2A). KIM-1 is a transmembrane protein not detectable in normal kidney tissue, nevertheless, it is expressed at very high levels after ischemic injury [27]. This molecule has been proposed as a sensitive biomarker of acute renal tubular injury [28], and its mRNA expression associates with interstitial fibrosis and tubular atrophy in kidney transplant recipients [29]. As expected from our previous studies [30], IRI-dependent induction of *KIM-1* mRNA expression was increased after IRI in the ischemia-reperfusion treated kidneys. This increase was prevented by morphine and fentanyl preconditioning (Figure 2B). In addition, the morphine group showed a more evident reduction in the expression of *KIM-1* compared to fentanyl group (*p* = 0.029). Overall, these results indicate that opioid preconditioning resulted in renal protection after IRI.

### 2.2. Opioids Stimulated HIF-1α Expression in the Kidney

**Figure 3:**
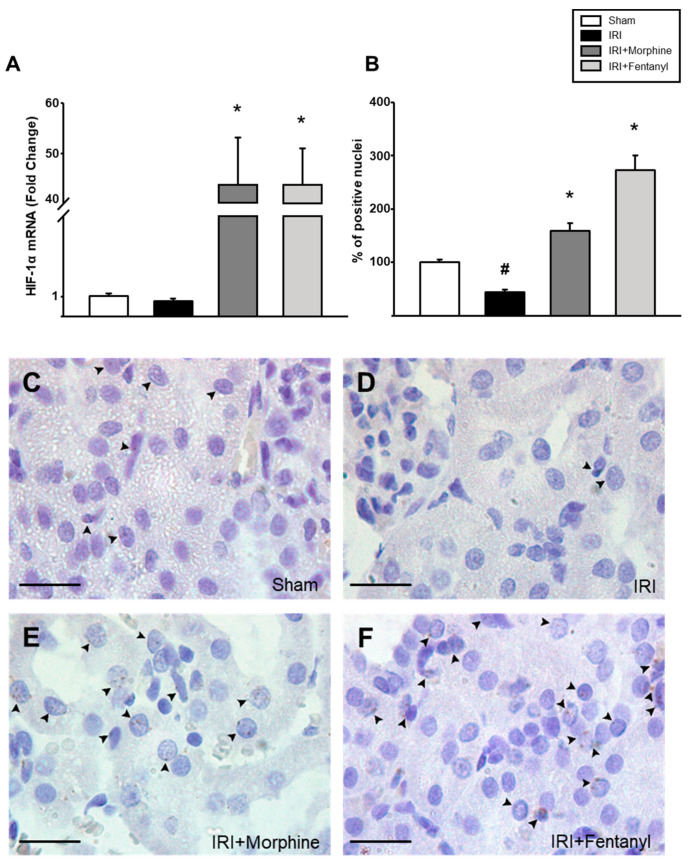
Opioid preconditioning promoted HIF-1 expression. (**A**) *HIF-1α* mRNA expression was analyzed by real-time PCR in the ischemia-reperfusion treated kidneys. Immunohistochemistry was performed in the ischemia-reperfusion treated kidney slides and the number of positive signal nuclei was quantified at 100× (**B**) from sham group (**C**), IRI (**D**), morphine- (**E**) and fentanyl-preconditioned animals (**F**). The average count of positive nuclei in the sham group was set as 100%. Sham group is represented by the white bars, IRI by the black bars, IRI + morphine by the dark gray bars, and IRI + fentanyl by the light gray bars. Scale bars represent 20 µm. Values are means ± S.E.M. (*n* = 6). # *p* < 0.05 vs. sham, * *p* < 0.05 vs. IRI.

HIF-1α is a transcription factor and a master regulator of adaptive responses to hypoxia [10,31]. Hence, we decided to measure the effect of opioid preconditioning on HIF-1α expression in the ischemia-reperfusion treated kidneys. The IRI group did not show a significant difference in the expression of the *HIF-1α* gene when compared to the sham group (0.773-fold ± 0.121 vs. IRI, *p* = 0.318; Figure 3A). Nevertheless, *HIF-1α* mRNA expression was strongly enhanced after morphine (43.730-fold ± 9.380) and fentanyl (43.796-fold ± 7.063) preconditioning, when compared to that of the IRI group (*p* = 0.001, see Figure 3A). Moreover, immunohistochemical analysis revealed an increase in HIF-1α expression (Figure 3B), observed as a dotted pattern located almost entirely inside the nucleus (Figure 3C–F).

### 2.3. Intrarenal Expression of VEGF and VEGF-R2 Was Modified by Opioid Preconditioning

**Figure 4:**
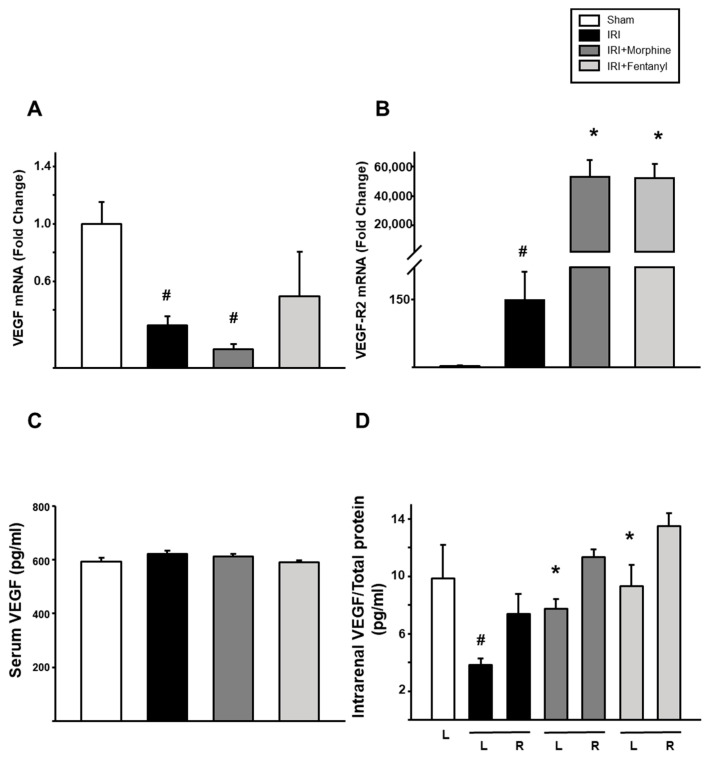
Opioid preconditioning modulated VEGF an VEGF-R2 expression. Quantitative RT-PCR evaluation of mRNA levels of *VEGF* (**A**) and *VEGF-R2* (**B**) in the ischemia-reperfusion treated left kidneys. Circulating (**C**) and intrarenal (**D**) levels of VEGF in the ischemia-reperfusion treated left kidneys (L) and the nontreated right ones (R). Levels were normalized using total protein quantification. Sham group is represented by the white bars, IRI by the black bars, IRI + morphine by the dark gray bars, and IRI + fentanyl by the light gray bars. Values are means ± S.E.M. (*n* = 6). # *p* < 0.05 vs. sham left (L) kidney. * *p* < 0.05 vs. IRI left kidney.

Next, we evaluated whether opioid preconditioning affected the expression of VEGF and its type-2 receptor (VEGF-R2) in the ischemia-reperfusion treated kidneys. *VEGF* mRNA in the left kidney showed a marked reduction after IRI (0.294-fold ± 0.0647 vs. sham, *p* < 0.001). Morphine preconditioning group showed a significant reduction (0.128-fold ± 0.0376, *p* = 0.01) compared to the sham group, but was not different to IRI group (*p* = 0.093). Moreover, fentanyl did not modify its expression level (*p* = 0.171 compared vs. sham) as shown in Figure 4A. *VEGF-R2* mRNA increased 146.890-fold after IRI when compared to that of the sham group (*p* = 0.001, Figure 4B). Remarkably, morphine and fentanyl preconditioning strongly influenced *VEGF-R2* mRNA expression after IRI, reaching 53,177.573-fold ± 10,945.481 and 52,153.628-fold ± 9505.615, respectively (*p* < 0.001, Figure 4B). When circulating VEGF was evaluated, similar levels were observed between the four groups (Figure 4C). Intrarenal protein levels of VEGF were then evaluated in whole organ extracts from the ischemia-reperfusion treated left kidneys and the untreated right kidneys, independently. As shown in Figure 4D, regardless of treatment, VEGF concentration in the right kidney lysate was similar. In the IRI group, VEGF levels in the left kidney were dramatically reduced (3.84 ± 0.443 pg/mL) as compared to those of the sham group (9.84 ± 2.363 pg/mL, *p* = 0.01). Remarkably, this reduction in the ischemia-reperfusion treated left kidney was prevented by morphine and fentanyl preconditioning, reaching levels like those of the sham group (9.29 ± 1.489 pg/mL and 7.73 ± 0.666 pg/mL, respectively, *p* < 0.001. See Figure 4D).

### 2.4. Opioids Promoted the Expression of Molecules Associated with Vessel Formation

**Figure 5:**
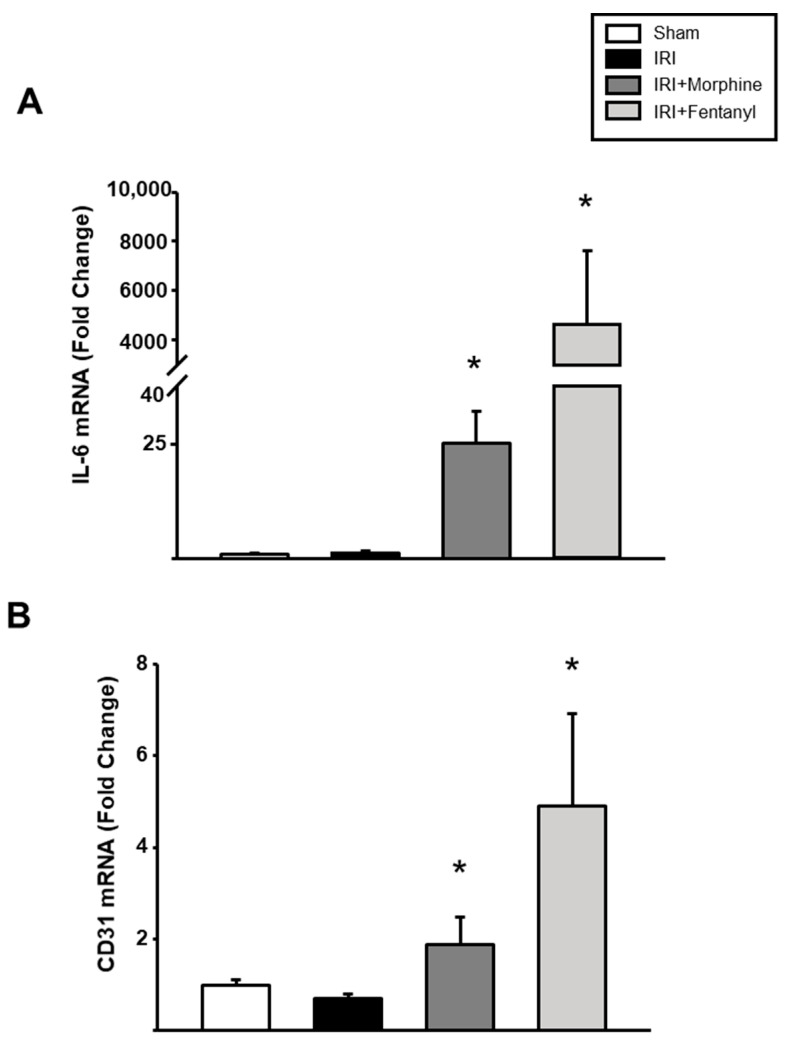
*IL-6* and *CD31* expression levels after IRI injury. Quantitative RT-PCR evaluation of renal expression of *IL-6* (**A**) and *CD31* (**B**) after ischemia-reperfusion with or without opioid preconditioning. HPRT was used as a housekeeping gene for normalization. Sham group is represented by the white bars, IRI by the black bars, IRI + morphine by the dark gray bars and IRI + fentanyl by the light gray bars. Values are means ± S.E.M. (*n* = 6). * *p* < 0.05 vs. IRI.

Then, we measured the expression of *IL-6* and *CD31*, two molecules that influence new blood vessel formation. We found a statistically significant increase in the mRNA expression levels of both genes in the ischemia-reperfusion treated left kidneys after morphine or fentanyl preconditioning (*p* < 0.001, Figure 5A,B).

### 2.5. Opioid Preconditioning Prevented the Increase in IRI-Induced Cathepsin D Expression

**Figure 6:**
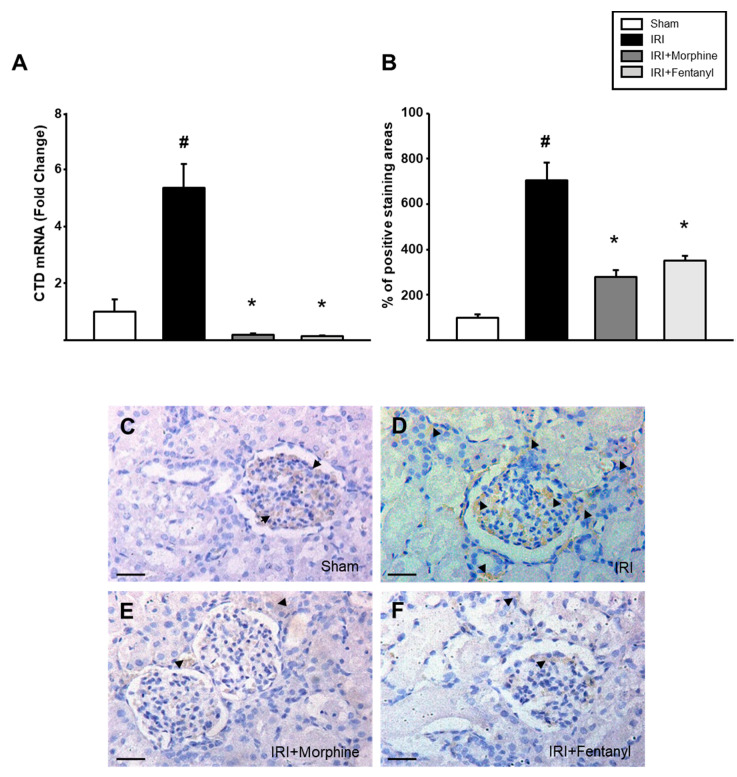
Opioid preconditioning reduced CTD expression. (**A**) *CTD* mRNA expression was examined by real-time PCR. Immunohistochemistry was executed in the ischemia-reperfusion treated left kidney and areas with positive staining (**B**) from the sham group (**C**), IRI (**D**), morphine- (**E**) and fentanyl-preconditioned rats (**F**) were quantified. Sham group is represented by the white bars, IRI by the black bars, IRI + morphine by the dark gray bars and IRI + fentanyl by the light gray bars. The average count of positive staining areas in the sham group was set as 100%. Scale bars represent 50 µm. Values are means ± S.E.M. (*n* = 6). # *p* < 0.05 vs. sham, * *p* < 0.05 vs. IRI.

CTD is a lysosomal protease that negatively contributes to the development and progression of AKI after IRI [32]. Thus, we decided to measure CTD expression and to determine its intrarenal localization in our model. Real-time PCR analysis revealed a 5.355-fold ± 2.810 increase in *CTD* mRNA expression in the ischemia-reperfusion treated left kidneys from the IRI group when compared to that of the sham group (*p* = 0.007). Conversely, opioid preconditioning prevented this increase, as the levels of *CTD* expression fell to basal levels (0.182 ± 0.0427 with morphine and 0.138 ± 0.0341 with fentanyl, *p* = 0.001; Figure 6A). Similarly, we noted a reduction in CTD signal detected in immunohistochemistry in the ischemia-reperfusion treated left kidneys both in morphine- and fentanyl-preconditioned groups (Figure 6C–F), as shown by the signal-positive area quantification measured by using ImageJ (*p* = 0.002, Figure 6B).

## 3. Discussions

Although microvascular rarefaction is one of the most controversial pathological events after IRI [33], there is limited evidence of strategies able to reduce it. This study supports a role for molecules involved in vascular repair responses in the mechanisms of renal protection mediated by opioid preconditioning.

Our study confirms that preconditioning using opioids provided protection to the kidney against ischemia-reperfusion injury. When analyzing kidney function (creatinine and *KIM-1* mRNA [28]), we found a notable decrease in the groups with pharmacological stimulus, compared to the IRI group. Participation of opioids in protection against surgical models has been extensively studied in organs such as the heart [34] and brain [35]. In kidney transplantation, similar molecules could be involved in the shown protection. Our results also demonstrate that morphine and fentanyl modulated the expression of proteins that promote angiogenesis and kidney tissue repair. We found changes in mRNA and protein expression of HIF-1α, VEGF, VEGF-R2, IL-6, CD31 and CTD after IRI, as well as influence of opioid preconditioning in AKI prevention.

Although kidney transplantation is the best therapeutic option for patients suffering ESRD [36], the transplanted organ must still overcome IRI-associated damage produced during and after the surgical process [37]. The challenge for the graft is to reach tissue reparation. Unfortunately, incomplete repair of kidney endothelium contributes to progressive organ dysfunction [38]. An impaired vascular response occurs as a reaction to decreased renal perfusion and leads to the release of reactive oxygen species (ROS) and cytokines resulting in renal vasoconstriction and chronic capillary loss [5]. Vascular rarefaction is an IRI mechanism implicated in tissue restoration delay. The loss of renal microvasculature intensifies renal hypoxia and contributes to damage progression [38]. Hence, promoting angiogenesis represents a novel therapeutic target to protect renal vasculature [39,40]. Nevertheless, the mechanisms remain poorly understood.

Hypoxia triggers several adaptive responses mostly orchestrated by HIF-1α, a transcription factor expressed in tubular and glomerular epithelial cells [41]. A large amount of evidence suggests that the expression of HIF-1α plays a protective role in kidney function [42,43,44]. Some authors have evaluated strategies to induce HIF-1α activity in the kidney [45,46]. However, the role of increased HIF-1α in kidney physiology seems to be controversial. Some studies show that activation of HIF-1α significantly reduces ischemic AKI by modulating the expression of HIF target genes [42]. On the other hand, a dysregulated and continued activation of HIF-1α promotes renal fibrosis [47,48]. Clinical studies suggest that the expression of a genetic variant of HIF-1α characterized by more stability in the protein associates with adverse outcomes in AKI [49].

After IRI, the hypoxic environment and HIF-1α stimulate the cells to secrete VEGF. VEGF mRNA is broadly expressed in the kidney [50]. Still, the mechanisms by which it exerts its physiological and pathological functions in this organ are diverse and complex. Our results show that the influence of the ischemic stimulus reduced the bioavailability of VEGF, consistent with other studies suggesting the reduction of this factor [51]. This result advises a risk to adequate functional maintenance of renal vasculature. The decrease in VEGF has been previously associated with the development of hypertension and significant loss of podocytes, aggravating the progression of kidney damage [52,53,54]. Indeed, some authors propose that VEGF therapy could promote renal microvascular repair to reverse rarefaction responses [55,56]. Here, we show that opioid preconditioning promoted VEGF protein expression directly in the left ischemic kidney, indicating activation of proangiogenic responses aimed at reducing microvascular loss. This finding correlated with an improvement in renal function observed after IRI in morphine- and fentanyl-preconditioned animals. VEGF activates signal transduction networks through VEGF-R2 to start angiogenesis [57]. Although VEGF mRNA expression was not increased under the stimulus of opioids, we found a significant increase in intrarenal VEGF Receptor-2 mRNA expression. This increase, mediated by both morphine and fentanyl, may represent a compensatory response to the decrease in VEGF mRNA expression. Interestingly, we also found increased expression of markers associated with new vessel generation, such as CD31 and IL-6, under the influence of both opioids. However, we only evaluated the angiogenic molecules at one time, and it could be interesting to explore expression changes in a chronic scenario.

CTD is the major lysosomal protease implicated in AKI progression via apoptosis activation [58]. We report an increase in CTD expression after IR, a finding in accordance with a previous paper [59]. Under pharmacological preconditioning stimulation, we found a complete ablation of CTD gene and protein expression. A decrease in CTD activity has been associated with a reduction in fibrosis and chronic IRI [59]. Our results show a novel use of opioids in the reduction of deleterious responses to the kidney, supporting their perioperative use as a potential therapeutic strategy to promote repair responses after transplantation and AKI.

This study supports the importance of the use of pharmacological agents such as opioids in interventions that present a period of ischemia such as transplantation. This new use of opioids represents a promising and effective intervention to recover renal function after IRI. To date, microvascular rarefaction processes are poorly understood. However, commonly used clinical strategies to prevent the development of kidney damage are a therapeutic advantage that should be applied based on experimental results. The stimulation of proangiogenic molecules to prevent IRI is a field that remains of broad interest.

Opioids have become a mainstay of modern anesthesia, and recent research has shown that the role of opioids goes beyond providing surgical analgesia. Currently, morphine and fentanyl are used effectively for anesthesia/analgesia regimens in almost all kinds of surgeries. It is known that these schemes help provide adequate antinociception and block the adrenergic response to pain, thus helping to maintain an adequate hemodynamic state in the intraoperative period, avoiding an increase in heart rate as well as blood pressure, imperative conditions in a kidney transplantation procedure.

Opioid preconditioning can be a clinically reasonable low-cost strategy that may be used in patients undergoing kidney transplantation, reducing complications, costs and hospital stay.

Our study has some potential limitations. It is largely known that the translation of mouse results to humans is difficult, and the transplantation area of research is not an exception. In this regard, the common practice of using a combination of drugs during transplantation surgeries to achieve analgesia and anesthesia makes it even more complicated. However, our work will help to provide a basis in the transplantation area for the safe use of pharmacological strategies for kidney protection.

Likewise, the study of the effects of opioids on the kidney demonstrates that they can be widely used in kidney patients and can be added to the currently established clinical criteria.

## 4. Materials and Methods

The methods were defined following ARRIVE guidelines for reporting animal research [60].

### 4.1. Experimental Animals

All experiments were conducted following institutional and federal regulations (NOM-062-200-1999) for animal wellness. This protocol received approval by the Local Health Research Committee and IACUC under registration number CI 14-039-114. Experimental surgery was made using male Wistar rats. The animals were housed under the strict care and handle of an experienced veterinary in the CIBO animal facilities. Animals had access to water and were fed with standard rodent chow available ad libitum. Habitat conditions were supplied always respecting 12-12 h of light and dark cycles, controlled temperatures and humidity according to the species.

### 4.2. Study Design

The study design is described in Figure 1. In brief, the animals were randomly separated into four experimental groups: sham (*n* = 6), unilateral ischemia-reperfusion (IR, *n* = 6), IR + morphine (*n* = 6), and IR + fentanyl (*n* = 6). Twenty-four rats weighing 200–250 g were prepared using xylazine (8 mg/kg, intraperitoneally (i.p.)) for sedation and analgesia, and ketamine (100 mg/kg, intraperitoneally) for maintaining anesthesia. Body temperature was always preserved at about 37 °C.

### 4.3. Experimental Procedures

We chose unilateral renal ischemia reperfusion injury as a model of acute kidney injury. An incision was made in the abdominal medium line to expose the kidney pedicle. Left renal hilum was dissected and then occluded with a microvascular clamp for 45 min. Renal hilum was released, and the surgical wound was closed according to the correct anatomical planes. Finally, the animals were placed in a cage for a period of 24 h for reperfusion (Figure 1). Preconditioning opioids (0.5 mg/kg morphine, 10 µg/kg fentanyl) were administered i.p. 1 h before the surgical procedure. Opioid doses were calculated based on previous reports [61,62]. Sham group surgery was performed doing a small incision and simple exploration of the renal hilum without occlusion. Once the surgery protocol was completed, the animals were kept under observation in an incubator at 37 °C until their full recovery. At the end of reperfusion, a blood sample and both kidneys were obtained from each rat for biochemical and immunohistochemical assays.

### 4.4. Real-Time PCR

The whole ischemia-reperfusion treated left kidneys were used to obtain mRNA with a RiboZol RNA extraction reagent (AMRESCO, VWR life science, Radnor, PA, USA). Reverse transcription was done with a QuantiTect Reverse Transcription Kit (Qiagen, Hilden, Germany). Quantitative real-time PCR was performed using the ready-to-use mix of enzyme and SYBR Green qEvaGreen (qARTA Bio, Inc., Carson, CA, USA) in a Lightcycler 96 analyzer (Roche Molecular Systems, Inc., Pleasanton, CA, USA). The expression of kidney injury molecule 1 (*KIM-1*), *IL-6*, *CD31*, *HIF-1α*, *VEGF*, *VEGF-R2* and *CTD* genes in the ischemia-reperfusion treated left kidney was determined using specific oligonucleotides (Table 1). The amplification conditions were as follows: 10 s at 95 °C, 30 s at primer-specific annealing temperature and 30 s at 72 °C for 40 cycles. Relative expression for each gene was quantified using the 2-ΔΔCt threshold method. Hypoxanthine phosphoribosyl transferase (*HPRT*) mRNA was used as a housekeeping gene.

### 4.5. Luminex

To evaluate circulating levels of VEGF, we used a rat premixed magnetic Luminex assay kit (R&D Systems, Minneapolis, MN, USA). Additionally, we measured intrarenal VEGF in whole lysates of the ischemia-reperfusion treated left kidneys using the right kidneys (without treatment) as controls. The Luminex kit was evaluated on a Luminex 200 (Luminex Corporation, Austin, TX, USA). Tests were performed following the fabricant instructions.

### 4.6. Immunohistochemistry

The ischemia-reperfusion treated left kidneys were fixed with 10% paraformaldehyde, processed and immersed in paraffine blocks. Microtome sections of 5 µm were obtained. Two commercially obtained antibodies were used: HIF-1α (1:100; Santacruz, CA, USA; sc-13515) and CTD (1:100; Abcam, Cambridge, UK; ab6313). Kidney sections were processed as described previously [63], with some modifications. Briefly, antigen retrieval was performed at high pressure and 100 °C in a sodium citrate buffer (pH = 6) for 5 min. Slides were incubated for 15 min with 0.3% H_2_O_2_, permeabilized for 10 min in 0.05% Triton X-100 in PBS buffer, blocked for 3 h in PBS, 1% BSA, 5% normal goat serum and washed for 5 min in 0.1% Triton X-100 in PBS. Incubation with the specified antibodies was performed overnight in the same solution. After being washed three times with a solution of 0.1% Triton X-100 in PBS, the sections were incubated in a solution containing the secondary antibody coupled to horseradish peroxidase (Goat anti-mouse HRP conjugate, 1:200, Enzo, Farmingdale, NY, USA) for 2 h at room temperature. Specimens were incubated with diaminobenzidine tetrahydrochloride, 0.03% High def DAB chromogen/substrate set (Enzo, Farmingdale, NY, USA) for color development. Finally, a counterstain with hematoxylin for 15 min was performed. Images were acquired using a Leica Optical microscope under 100x for HIF-1 and 40x objective for CTD and processed identically for brightness and color balance using Photoshop 13 (San Jose, CA, USA). Briefly, ten fields were randomly chosen in renal cortex slides. For HIF-1, cells presenting signals inside the nucleus were counted as positive. The average of HIF-1 count in the sham group was set as 100%. Quantification was not blinded and performed using ImageJ software by two different users (US National Institutes of Health, Bethesda, MD, USA) [64].

### 4.7. Statistical Analysis

Data are presented as mean ± standard error of the mean (S.E.M.). The analysis was performed using the Shapiro–Wilk test for normality, followed by ANOVA test for comparison between groups. Sigma Stat and Sigma plot software (Systat Software, San Jose, CA, USA) were used. The threshold for statistical significance was set at *p* < 0.05.

## 5. Conclusions

Our results show that opioid preconditioning protected the kidney from IRI through modulation of tissue repair responses. HIF-1, VEGF and other angiogenic factors are important targets to consider when establishing strategies aimed at ameliorating ischemic damage in pathophysiological and surgical processes in which renal ischemia-reperfusion occurs.

## Figures and Tables

**Table 1:** List of oligonucleotides used to evaluate KIM-1, IL-6, CD31, HIF-1α, VEGF, VEGF-R2 and CTD gene expression.

Target Gene	Sequences (5′-3′)		Annealing Temp. (°C)
***KIM-1***	F-TCCTGTGGGATTCATGCAGT	R-GCAGGAGGCCTGAAATGAAG	53
***IL-6***	F-TGAGAAAAGAGTTGTGCAATGG	R-GCATCATCGCTGTTCATACAAT	51
***CD31***	F-TTGTGACCAGTCTCCGAAGC	R-TGGCTGTTGGTTTCCACACT	54
***HIF-1α***	F-GCAACTGCCACCACTGATGA	R-GCTGCTTGAAAAAGGGAGCC	54
***VEGF***	F-GGCCTCTGAAACCATGAACT	R-TGCTCCCCTTCTGTCGTG	53
***VEGF-R2***	F-TTTTGGCAAATACAACCCTTC	R-AGATTACTTGCAGGGGACAGA	53
***CTD***	F-CCGTCGGACTATGACGGAAG	R-ACAGCTCCCCGTGGTAGTAT	60.2
